# Assessing compliance with smoke-free laws in Purbakhola Rural Municipality, Nepal: A cross-sectional observational study

**DOI:** 10.1371/journal.pgph.0005499

**Published:** 2026-05-11

**Authors:** Bhakta Bahadur KC, Ananda Bahadur Chand, Amit Bam, Tara Singh Bam

**Affiliations:** 1 Ministry of Health and Population, Kathmandu, Nepal; 2 Action Nepal, Kathmandu; 3 Vital Strategies, Asia Pacific Office, Singapore; University of Health and Allied Sciences, GHANA

## Abstract

Despite Nepal’s comprehensive tobacco control legislation, evidence on its implementation at the local level, particularly in rural areas, remains limited. This study assessed compliance with smoke-free laws in public places of Purbakhola Rural Municipality, Palpa, Nepal. A cross-sectional observational survey was conducted from May 7 to May 16, 2025, across all six wards of the municipality. We assessed 219 public places from 11 categories. Compliance with smoke-free provisions was measured using an adapted observational checklist covering six indicators: active smoking, no-smoking signage, designated smoking areas, ashtrays, cigarette butt litter and tobacco advertisement. Descriptive statistics estimated compliance and exploratory logistic regression examined associations between observed active smoking and environmental factors, including the presence of ashtrays and no-smoking signage. Compliance with smoke-free provisions was high in public institutional settings, including health facilities (100.0%), educational institutions (100.0%), and government offices (88.8-100.0%). In contrast, substantial non-compliance was observed in the hospitality sector, where only 45.0% of restaurants were free from indoor smoking and 37.5% had no visible ashtrays. A critical gap was the lack of systemic compliant no-smoking signage, even in high-compliance settings, with 54.5% of health facilities non-compliant indoors and 100.0% of government offices non-compliant outdoors. The presence of ashtrays was the strongest predictor of smoking, with indoor ashtrays associated with dramatically higher odds of observed smoking. In contrast, no statistically significant association was found for signage, although no smoking was observed in venues with indoor signage. There is a significant implementation gap in smoke-free law compliance particularly in the hospitality sector. The findings underscore the urgent need for strengthening enforcement, ensuring the removal of ashtrays—a major environmental cue for smoking—and promoting the universal installation of standardized no-smoking signage to align practice with policy and protect public from secondhand smoke.

## Introduction

Tobacco use remains one of the world’s most significant public health threats, responsible for over 8 million deaths annually, including approximately 1.6 million deaths among non-smokers exposed to secondhand smoke (SHS) [1–3]. The burden of tobacco-related disease is disproportionately borne by low- and middle-income countries like Nepal, where tobacco smoking prevalence is 46.3% among men and 11.6% among women, contributing to over 39,000 deaths yearly [4–7]. Exposure to SHS poses serious health risks to non-smokers, including increased risks of heart disease, lung cancer, and respiratory illnesses, highlighting the importance of effective smoke-free public policies to protect non-smokers [8].

In response, Nepal ratified the World Health Organization Framework Convention on Tobacco Control (WHO FCTC) in 2006 [9]. The WHO FCTC Article 8 and its guidelines establish 100% smoke-free environments as the best practice for protecting citizens from SHS [10]. Similarly, the MPOWER-the WHO’s technical assistance package of evidence-based policies for tobacco control-also identifies the adoption of 100% smoke-free policies as a critical strategy to reverse the tobacco epidemic. Consistent with these recommendations, Nepal enacted the Tobacco Product (Control and Regulatory) Act and Rule in 2011, which prohibit smoking in all public places and workplaces [11–13]. The act classifies the public places into multiple categories, including indoor and outdoor areas of government offices, health facilities, educational institutions, hospitality venues, etc. and requires the display of standardized no-smoking signage.

Under Nepal’s federal governance system, responsibility for implementing and enforcing tobacco control laws rests primarily with local governments. A cabinet decision in January 2017, designated the chief executive officer of every local level as tobacco inspector and global studies reinforce that the role of local governments is vital for the effective implementation of tobacco control laws [14,15]. However, despite the existence of this institutional framework, compliance and enforcement of these smoke-free public places remain challenges, particularly at the local levels [16].

Emerging evidence indicates substantial gaps in compliance with the smoke- free legislation at the municipal level, contributing to continued exposure to secondhand smoke in public places [17,18]. Existing studies in Nepal have largely focused on urban centers or national-level assessments and often fail to capture the realities of enforcement in rural settings [17,19]. Moreover, limited attention has been given to environmental cues, such as ashtrays and signage which may influence smoking behavior and compliance.

To address these gaps, this study systematically assessed compliance with smoke-free laws across multiple categories of public places in Purbakhola municipality of Nepal. By identifying setting-specific compliance gaps and environmental factors associated with observed smoking behavior, the study aims to generate locally relevant evidence to inform municipal enforcement strategies and contribute to the broader understanding of smoke-free policy implementation in federal contexts.Bottom of Form

## Methods

### Study design and setting

A cross-sectional observational study was conducted from May 7 to May16, 2025, in all six wards of Purbakhola Rural Municipality where the population is 16,052. This rural municipality is located in a hilly region of Lumbini Province of Nepal where diverse ethnic groups live. The study protocol and tools were adapted from the Johns Hopkins Bloomberg Initiative’s guide for assessing compliance with smoke-free laws [20].

### Characterization of public places:

Public places were categorized based on their primary function, owner and typical user population. Health facilities included health posts, Ayurved Aushadhalaya, community health units, and health section office. Government offices comprised one rural municipality administrative office, six ward offices, and one post office, and one Nepal Electricity Authority office. Educational institutions included primary and secondary schools and higher secondary colleges. Restaurants and hotels ranged from small roadside eateries (*khaja ghar*) to registered lodges, generally characterized by moderate to high daily foot traffic. Retail Goods Outlets (Groceries/Shops) included grocery stores, hardware shops outlets frequently visited by the general public. Public Transport Vehicles and terminals included locally operating buses and jeeps, while terminals referred to designated bus or jeep stops.

### Sampling and sample size

A comprehensive list of public places was obtained from the Purbakhola rural municipality office and verified through field visits. The minimum required sample size was initially calculated as 576 public places using a 50% compliance rate, 5% margin of error, 95% confidence level, and a design effect of 1.5 via OpenEpi (Version 3). However, the total number of available public places in the study area was found to be less than this calculated requirement. Therefore, all 219 identified public places were included in the study.

### Data collection and tool

Observations were categorized into indoor and outdoor areas. Outdoor observations focused on the immediate premises of the venue; however, for schools, colleges, health facilities, the assessment included the area within a 100-meter radius to monitor compliance in accordance with national tobacco control regulations.

To minimize social desirability bias, enumerators conducted brief observations lasting 7–10 minutes at each venue without disclosing the specific focus on tobacco control compliance. Observations were carried out during peak business hours and relied primarily on direct visual observation. Nonetheless, the potential for residual social desirability bias is acknowledged as a study limitation.

The checklist consisted of three main sections. The first section, location and visit information, documented facility or site characteristics including name, address, venue type, number of buildings, time of visit. The second section, indoor and outdoor observations, which employed yes/no questions to document evidence of smoking, no-smoking signage, ashtrays, and designated smoking areas. The third section outlined a permission protocol, for obtaining verbal consent from site managers for photography and entry permission. The tool assessed the following six compliance indicators [20]:

1. Absence of active smoking
2. Presence of no-smoking signage (e.g., written as Smoking Prohibited Zone).
3. Absence of smoking aids (e.g., ashtrays, lighters, matches).
4. Absence of designated smoking zone.
5. Absence of cigarette butt litter.
6. Absence of tobacco product advertisements with rate list displayed or posted.

Photographic evidence was collected to support observations. The supervisor validated data collection by accompanying teams to over 25% of sites as well as by checking data.

Data were entered using a double-entry system to ensure accuracy and analyzed using IBM SPSS version 31. Public places were categorized into 11 types (e.g., health facilities, educational institutes, restaurants, government offices etc.) and descriptive statistics were computed to determine compliance rates for each indicator across these categories of public places.

### Data entry and analysis

An exploratory univariable logistic regression analysis was conducted to assess associations between observed smoking (binary outcome) and selected environmental factors, including visible indoor ashtrays, visible outdoor ashtrays, and the presence of no-smoking signage indoors and outdoors. Odds ratios (ORs) with 95% confidence intervals (CIs) were estimated. Statistical significance was set at p < 0.05. This analysis was hypothesis-generating and not intended to establish causality.

### Ethical considerations:

This study was based on observations in public places and did not collect personal information. Verbal permission was obtained from venue managers or owners prior to observation and photography. Photographs were limited to environmental indicators (e.g., signage, ashtrays) and did not capture identifiable individuals. All collected data were kept confidential and used solely for research purposes. The study complied with ethical principles for observational research in public settings. Formal ethical review was not required as per national guidelines for non-human subject research.

### Operational definitions

#### Compliance.

It is the degree to which the Tobacco Product (Control and Regulatory), Act 2011 Nepal is complied with.

#### Smoke-free legislation.

The law that prohibits smoking in a specified area as per the Tobacco Product (Control and Regulatory) Act 2011 Nepal.

#### Public Places.

All the places that are defined as public places in the Tobacco Product (Control and Regulatory), Act 2011 Nepal.

#### Outdoor.

The outer premises of public places but includes a 100 meter around the school, health facilities, senior citizen home, child shelter places as per Tobacco Product (Control and Regulatory), Act 2011 Nepal.

#### Smoking.

Inhaling or exhaling the smoke of tobacco and also includes keeping or controlling any flamed tobacco products.

#### Active smoking.

Active smoking in a public place was marked as present if anyone was seen smoking during the researcher’s visit at the public place being observed for the study.

#### No-smoking signage.

According to Tobacco Product (Control and Regulatory), Act 2011 Nepal every public place shall arrange to display a caution notice **“**Smoking Prohibited Area, It Is a Punishable Offence**”** in Nepali language at the entrance and in one or more places inside a public place. The size of a caution notice board in a public place shall be at least 40 centimeters * 20 centimeters. Every public place shall arrange to display the caution notice in red letters against a white background or in white letters against a red background with no-smoking signage.

#### Cigarette butts, bidi ends, or ashes.

Cigarette butts, bidi ends, or ashes were marked as present as evidence of previous smoking in a particular public place if any of these were noticed during the researcher’s visit at the public place being observed for the study.

#### .Smoking aids.

According to Tobacco Product (Control and Regulatory), Act 2011 Nepal, no smoking aids such as ashtrays, matchboxes, or lighters can be kept in public places.

## Results

### Type and number of public places observed

A total of 219 public places were observed across 11 categories. The largest numbers of sites were retail goods outlets (n = 52), Restaurant/ *Chamena Griha (*Canteen*)*/*Khaja Ghar (*Eateries*)* (n = 40), and educational institutes (n = 33), while public transport vehicles and terminals (n = 6) and other sites (n = 6) were the least observed (Table 1).

**Table 1:** Type and Number of Public Places Observed.

Categories of Public Places	Number observed	Percentage
Health Facilities	11	5.0%
Government Office Building	8	3.7%
Educational Institutes	33	15.1%
Private Office Building (Bank etc)	10	4.6%
Place of Worship	27	12.3%
Retail Goods Outlets (Groceries/Shops)	52	23.7%
Fitness Centers/Sports Facilities/Recreation Parks	14	6.4%
Hotel/Lodge	12	5.5%
Restaurant/*Chamena Griha* (Canteen)/*Khaja Ghar* (Eateries)	40	18.3%
Public Transport and Terminals	6	2.7%
Others	6	2.7%
Total	219	100%

### Indoor compliance with smoke-free laws across different public places

A clear disparity in compliance was evident between public institutions, e.g., health facilities, college, government buildings and hospitality settings. While health facilities, educational institutions, and government offices showed a high level of observed compliance in prohibiting active smoking, designated smoking areas, and tobacco-advertisements, hospitality venues demonstrated marked deficits. The prevalence of active indoor smoking was high in restaurants (55.0%) and hotels (41.7%), corroborated by the frequent presence of visible ashtrays. The most widespread violation, however, was the absence of compliant signage, which was observed in 54.5% of health facilities and all government offices (Table 2).

**Table 2:** Compliance with smoke-free laws for indoor observations across different public places.

Categories of Public Places	N	No active smoking (%)	No designated smoking area (%)	No ashtrays visible (%)	Signage displayed (%)	No cigarette butts (%)	No tobacco advertisement (%)
Health Facilities	11	11 (100.0)	11 (100.0)	11 (100.0)	5 (45.5)	11 (100.0)	11 (100.0)
Government Office Buildings	8	7 (87.5)	8 (100.0)	8 (100.0)	1 (12.5)	7 (87.5)	8 (100.0)
Educational Institutes	33	33 (100.0)	33 (100.0)	33 (100.0)	2 (6.1)	33 (100.0)	33 (100.0)
Private Office Buildings (Banks etc.)	10	10 (100.0)	10 (100.0)	10 (100.0)	0 (0.0)	10 (100.0)	10 (100.0)
Places of Worship	27	21 (77.8)	27 (100.0)	27 (100.0)	0 (0.0)	22 (81.5)	27 (100.0)
Retail Goods Outlets	52	41 (78.8)	52 (100.0)	45 (86.5)	1 (1.9)	45 (86.5)	48 (92.3)
Fitness Centers/Parks	14	13 (92.9)	14 (100.0)	14 (100.0)	0 (0.0)	9 (64.3)	14 (100.0)
Hotels/Lodges	12	7 (58.3)	11 (91.7)	5 (41.7)	0 (0.0)	5 (41.7)	12 (100.0)
Restaurants/*Chamena Griha* (Canteen)/*Khaja Ghar* (Eateries)	40	18 (45.0)	39 (97.5)	15 (37.5)	0 (0.0)	19 (47.5)	39 (97.5)
Public Transport/Terminals	6	5 (83.3)	6 (100.0)	6 (100.0)	0 (0.0)	4 (66.7)	6 (100.0)
Others	6	4 (66.6)	6 (100.0)	4 (66.7)	0 (0.0)	4 (66.7)	6 (100.0)

### Outdoor compliance with smoke-free laws across different public places

A high prevalence of smoking prohibitions was found outdoor areas of health facilities (90.9%) and schools (84.8%), in stark contrast to restaurants (42.5%). Notably, the absence of outdoor signage was nearly universal across all site types (Table 3).

**Table 3:** Compliance with smoke-free laws for outdoor observations across different public places.

Categories of Public Places	n	No Active Smoking (%)	Smoke-Free Area Established (%)	Compliant Signage Displayed (%)	No Ashtrays Visible (%)	No Cigarette Butts (%)	No Tobacco Advertisement (%)
Health Facilities	11	10 (90.9)	4 (36.4)	3 (27.3)	9 (81.8)	8 (72.7)	11 (100.0)
Government Office Buildings	8	7 (87.5)	1 (12.5)	0 (0.0)	8 (100.0)	5 (62.5)	8 (100.0)
Educational Institutes	33	28 (84.8)	33 (100.0)	0 (0.0)	33 (100.0)	30 (90.9)	33 (100.0)
Private Office Buildings (Banks etc.)	10	8 (80.0)	4 (40.0)	0 (0.0)	10 (100.0)	8 (80.0)	10 (100.0)
Places of Worship	27	18 (66.7)	10 (37.0)	0 (0.0)	27 (100.0)	20 (74.1)	27 (100.0)
Retail Goods Outlets	52	44 (84.6)	37 (71.2)	1 (1.9)	46 (88.5)	34 (65.4)	49 (94.2)
Fitness Centers/Parks	14	13 (92.9)	8 (57.1)	1 (7.1)	14 (100.0)	7 (50.0)	14 (100.0)
Hotels/Lodges	12	11 (91.7)	10 (83.3)	0 (0.0)	12 (100.0)	10 (83.3)	12 (100.0)
Restaurants/ (*Chamena Griha* (Canteen)/*Khaja Ghar* (Eateries)	40	23 (57.5)	31 (77.5)	0 (0.0)	33 (82.5)	13 (32.5)	39 (97.5)
Public Transport/Terminals	6	4 (66.7)	5 (83.3)	0 (0.0)	6 (100.0)	4 (66.7)	6 (100.0)
Others	6	6 (100.0)	6 (100.0)	0 (0.0)	5 (83.3)	3 (50.0)	6 (100.0)

### Factors associated with smoking behavior in public places

As shown in Table 4, the presence of visible ashtrays was strongly associated with smoking behavior. Venues with visible indoor ashtrays had 16 times higher odds of smoking being observed (OR=16.10, p < 0.001), while those with outdoor ashtrays had 7.5 times higher odds (OR=7.48, p < 0.001). No-Smoking signage showed no statistically significant associations, though venues with indoor signage demonstrated complete compliance (0% smoking prevalence).

**Table 4:** Factors Associated with Indoor and Outdoor Smoking in Public Places (N = 219).

Environmental Factor	Category	Indoor Smoking			Outdoor Smoking		
		n/N (%)	OR (95% CI)	P-value	n/N (%)	OR (95% CI)	P-value
Visible Ashtrays	No	21/178 (11.8)	Ref	–	37/203 (18.2)	Ref	–
	Yes	28/41 (68.3)	16.10 (7.2–35.8)	<0.001	10/16 (62.5)	7.48 (2.56–21.87)	<0.001
No-Smoking Signage	Present	0/10 (0.0)	–	0.122*	1/5 (20.0)	0.91	1.000
	Absent	49/209 (23.4)	–	–	46/214 (21.5)		

## Discussion

This study assessed compliance with smoke-free legislation in a Rural Municipality of Nepal, and found mixed and substantial variations across different categories of public places. Public institutional settings such as health facilities, educational institutions, and government offices showed high compliance with the prohibition of active smoking, designated smoking areas and tobacco advertisement. In contrast, hospitality sector (e.g., restaurants and hotels) showed markedly lower compliance with frequent indoor and outdoor smoking, visible ashtrays indicating routine rather than sporadic violations. Although outdoor compliance was generally higher than indoor compliance—particularly in institutional settings—the persistence of the same sectoral pattern suggests that enforcement gaps affect both environments. These findings highlight a critical implementation gap between smoke-free public place legislation and its enforcement at local levels particularly in rural Nepal.

These compliance patterns are consistent with national, regional, and international evidence showing that compliance is highest in public institutional settings and lowest in hospitality venues. Previous studies from Nepal have reported high compliance in government and health institutions but markedly low compliance in eateries and entertainment venues along with suboptimal national compliance and critically low levels of mandated signage, indicating systemic implementation failures [17,19,21]. While institutional and regulatory contexts differ across countries, similar enforcement challenges are evident in many settings. Comparable findings have been reported across South Asia, as well middle and high income countries where weak enforcement and limited monitoring continue to undermine smoke-free regulations in hospitality settings [22–31]. Under Nepal’s federal governance system, municipalities are primarily responsible for routine inspections and enforcement; however, limited regulatory capacity, infrequent inspections, and competing administrative priorities often constrain effective oversight in rural areas. As a result, violations may remain undetected or unaddressed. Moreover, small, privately operated hospitality businesses may prioritize customer satisfaction over regulatory compliance, contributing to the normalization of smoking and further weakening enforcement efforts.

The most widespread violation across all categories of public places was the absence of mandated no-smoking signage. Compliant signage was rarely observed in either indoor or outdoor areas, including in health facilities and government offices, where other indicators of compliance were relatively strong. This suggests that signage requirements are systematically neglected, even in government settings that otherwise adhere to smoke-free laws. The near-total absence of signage aligns with findings from Bangladesh, Guatemala, and Ethiopia, suggesting a recurring systemic failure [17,31,32]. Because the signage is vital for awareness and empowering public self-enforcement, its neglect is a critical operational weakness rather than a merely symbolic one [19,33].

This institutional neglect is compounded by normalized smoking behaviors and weak enforcement in the hospitality sector. Similar to trends reported in India and Bangladesh, the persistence of ashtrays and cigarette litter in Nepalese restaurants and hotels indicates a tacit acceptance of smoking, often driven by owners’ fears of losing customers [24,25]. These findings reinforce evidence from Pakistan and Nepal that legislation alone is insufficient [16,23]. To shift these social and cultural norms and change behavior, the legal framework must be supported by active monitoring, consistent enforcement, and the strict application of penalties.

Given the cross-sectional design and lack of control for potential confounders, the exploratory univariable regression analyses were intended to provide insight into environmental correlates of smoking behavior and to generate hypotheses rather than establish causal relationships. The findings shows that the presence of visible ashtrays-both indoors and outdoors- was the strongly associated with observed smoking with venues displaying ashtrays having markedly higher odds of observed smoking. Although causal inference is not possible given the cross-sectional design and univariable analyses, these findings suggest that ashtrays function as a powerful environmental cues that normalize and facilitate smoking- a conclusion supported by previous realist reviews [34]. Their presence may reflect acceptance of smoking by venue management and weaken smoke-free norms. Conversely, no statistically protective association was observed for no- Smoking signage. This findings may be attributed to very small number of venues displaying no-smoking signage. However, not a single instance of smoking was observed in any venue where signage was displayed suggesting a potentially meaningful protective effect.

These findings therefore have important policy implications. First, enforcement efforts must extend beyond public institutional settings to hospitality settings, where SHS exposure is likely to be greatest. Second, mandating no-smoking signage across all public venues is a simple, low-cost intervention with high potential impact as previous research has shown the presence of signage works as the means raising awareness of tobacco-free policies within institutions [35]. Finally, tobacco control should be integrated into routine municipal monitoring and enforcement structures, supported by public awareness campaigns aimed at shopkeepers and hospitality business owners and managers, particularly in rural areas where awareness of legal obligations may be limited.

## Strengths and limitations

This study is among the first to assess compliance with smoke-free laws in a rural municipality of Nepal, providing granular evidence across 11 categories of public places. The use of direct observation and photographic evidence strengthens the reliability of findings. However, the study was limited to one municipality and may not be generalizable to all rural areas of Nepal. Additionally, data collection during daytime business hours may underestimate non-compliance in restaurants and hotels, where evening smoking could be more prevalent.

Several additional limitations should be noted. Observations were conducted for a short duration (7–10 minutes per venue), which may not fully capture temporal variations in smoking behavior, particularly in hospitality venues during evening hours. Residual social desirability bias may have influenced the results. The exploratory regression analysis should therefore be interpreted as hypothesis-generating.

## Conclusions

This study demonstrates a significant implementation gap in smoke-free law compliance within Purbakhola Rural Municipality. While institutional settings like health facilities and schools showed near-perfect adherence, the hospitality sector exhibited persistence non-compliance with high rates active indoor smoking and widespread availability of ashtrays. The near universal absence of mandated no-smoking signage across public venues represents a critical systemic weakness in policy implementation.

To bridge this policy-practice divide, a targeted, multi-strategy approach is essential. Purbakhola Rural Municipality should immediately prioritize the universal installation of standardized signage across all public places, ensure the systematic removal of ashtrays and strengthen routine monitoring and enforcement mechanism particularly in hospitality setting. These efforts should be supported by targeted awareness campaigns for business owners and the general public and by integrating tobacco control programs into the municipal’s annual planning with a dedicated budget. Strengthening local implementation capacity is essential—to translate Nepal’s strong legal framework into effective public health protection from SHS in rural communities.

## Supporting information

S1 Checklist Adapted observational checklist used to assess smoke-free compliance. (DOCX)

S2 Checklist Inclusivity in global research. (DOCX)

S1 Dataset Observational data collected from public places in Purbakhola Rural Municipality. https://zenodo.org/records/19567952.
(CSV)
