# Supplementary material for: Assessing compliance with smoke-free laws in Purbakhola Rural Municipality, Nepal: A cross-sectional observational study
Source: PLOS Glob Public Health. 2026 May 11;6(5):e0005499. doi: 10.1371/journal.pgph.0005499 (PMC13160295; doi:10.1371/journal.pgph.0005499)
Supplement: S1 Checklist — (DOCX) [file pgph.0005499.s001.docx]

Assessing Compliance with Smoke-Free Laws in Purbakhola Rural Municipality, Nepal: A Cross-Sectional Observational Study

**Compliance Study Observation Tools**

Enumerator ID: ____________________ Date: ____________________

**PART 1. LOCATION INFORMATION**

| 1.Name of Location/facility: | | | |
| --- | --- | --- | --- |
| 2. Address: | | | |
| 3.Total number of building at location:  (*Include Q3 only if you will be observing multiple buildings within one location (e.g. a university)* | | | |
| 4.Type of location (choose only one) | | | |
| Types of public place | Description if needed | |  |
| 1. Health Facility | Health post, basic health centers, primary hospitals | |  |
| 1. Government Office Buildings | Offices of municipality, ward offices etc excluding health offices | |  |
| 1. Private Office Buildings | Banks, cooperative office | |  |
| 1. Education Facility (Primary & secondary) | Schools | |  |
| 1. Education Facility (College) | College | |  |
| 1. Place of Worship | Temples | |  |
| 1. Recreation park | Parks | |  |
| 1. Fitness center/Sports Faciluty | Fitness and football ground | |  |
| 1. Shops and Mall | Grocery, shops | |  |
| 1. Restaurant/*Chamena Griha*(Canteen)/*Khaja Ghar*(Eateries) |  | |  |
| 1. Hotel/Lodges |  | |  |
| 1. Public conveyance(Bus, Mini-Bus, Jeep) | Bus and public transport themselves | |  |
| 1. Bus /Jeep terminal | Bus park and bus station | |  |
| 1. Other |  |  |  |
| 5. Date of visit: ………/………../……………. | | | |
| 6.Data collector name/ code : | | | |
| 7.Time of entry to location: ……………am/pm  8.Time of departure: ……………am/pm | |  | |
| 9. Photo taken?  Yes  No | | | |
| 10.Result of observation Finished Not Finished *(Go to Q 11)* | | | |
| 11.If observation not finished, reason why: Data collector not allowed to enter building/location  Building/location out of business   Other | | | |

**PART II. OBSERVATION INFORMATION**

| **Observation Indoors** | |
| --- | --- |
| 12.Name or number of building: |  |
| 13. Is anyone smoking tobacco products indoors?   Yes  No |  |
| 14. Is there any designated smoking area indoors?   Yes  No  *(Choose “Yes” if you see a designated area, even if no one is smoking in it)* |  |
| 15. Are there any ashtrays visible indoors?   Yes  No |  |
| 16. Are there one or more no smoking signages in the venue/ location?   Yes  No |  |
| **OPTIONAL: Record any other comments regarding your indoor observation** | |

| **Observation Outdoors (inside location boundary)** |  |
| --- | --- |
| 17. Is anyone smoking within 10 meters from building entrances and windows?   Yes  No |  |
| 18. Is the location required to be smoke-free outdoors?   Yes *(Continue to Q 107)*   No *(Finish observation)* |  |
| 19. Is anyone smoking tobacco products anywhere outdoors at the location? (If there are no outdoor grounds at this location, choose “not applicable” and finish observation)   Yes  No  Not Applicable *(Finish observation)* | |
| 20. Are there one or more no smoking signage on the outdoor areas of the public places?   Yes  No | |
| 21. Are there any ashtrays/ashbin/ash cans visible outdoors at the location?   Yes  No | |
| **OPTIONAL: Record any other comments regarding your outdoor observation.** | |

**Appendix B: Verbal Permission to entry public places if it is indoor and also for Photography**

Part 1: Pre-Approach (Check Before Entering)

- ID badge is visible.
- I have identified the manager/person in charge.
- I am prepared to leave immediately if permission is denied.

Part 2: Verbal Permission Script (Check each step)

1. Introduction & Purpose

2. Clarify if Asked

If asked for details, say: "We are checking compliance with smoking laws, like signage and any evidence of smoking in Purbakhola, Palpa. I will not disturb your staff or customers."

3. Request Photography

The enumerator should say: "To ensure data accuracy, I need to take a few general photos of the area and signage. These are for internal use only and will not be published. Do I have your permission to take photos?"

PHOTOGRAPHY GRANTED

PHOTOGRAPHY DENIED

4. Confirm & Thank

The enumerator should say: "Thank you. To confirm, I have your permission to observe and take photos. I will be discreet."

Part 3: On-Site & Departure

- Conducted observation discreetly.
- Photos focused on signage/areas, not people.
- Upon leaving, say: "Thank you for your cooperation."

Part 4: Post-Visit Documentation

Final Status:

- FULL COOPERATION (Entry & Photos)
- PARTIAL COOPERATION (Entry, No Photos)
- NO COOPERATION- No taking data

Authorizing (Manager)Person: _________________ Title: _________________

Visit Time: Start: ________ End: ________
